# Virotheranostics, a double-barreled viral gun pointed toward cancer; ready to shoot?

**DOI:** 10.1186/s12935-020-01219-6

**Published:** 2020-04-23

**Authors:** Mohsen Keshavarz, Ailar Sabbaghi, Seyed Mohammad Miri, Abolhasan Rezaeyan, Yaser Arjeini, Amir Ghaemi

**Affiliations:** 1 The Persian Gulf Tropical Medicine Research Center, The Persian Gulf Biomedical Sciences Research Institute, Bushehr University of Medical Sciences, Bushehr, Iran; 2 Department of Influenza and Other Respiratory Viruses, Pasteur Institute of Iran, Tehran, Iran; 3 Department of Chemistry, Sharif University of Technology, Tehran, Iran; 4 Department of Medical Physics, School of Medicine, Iran University of Medical Sciences, Tehran, Iran; 5 Virology Department, School of Public Health, Tehran University of Medical Sciences, Tehran, Iran; 6 Department of Virology, Pasteur Institute of Iran, Tehran, Iran

**Keywords:** Oncolytic virotherapy, Measles virus, Molecular imaging

## Abstract

Compared with conventional cancer treatments, the main advantage of oncolytic virotherapy is its tumor-selective replication followed by the destruction of malignant cells without damaging healthy cells. Accordingly, this kind of biological therapy can potentially be used as a promising approach in the field of cancer management. Given the failure of traditional monitoring strategies (such as immunohistochemical analysis (in providing sufficient safety and efficacy necessary for virotherapy and continual pharmacologic monitoring to track pharmacokinetics in real-time, the development of alternative strategies for ongoing monitoring of oncolytic treatment in a live animal model seems inevitable. Three-dimensional molecular imaging methods have recently been considered as an attractive approach to overcome the limitations of oncolytic therapy. These noninvasive visualization systems provide real-time follow-up of viral progression within the cancer tissue by the ability of engineered oncolytic viruses (OVs) to encode reporter transgenes based on recombinant technology. Human sodium/iodide symporter (hNIS) is considered as one of the most prevalent nuclear imaging reporter transgenes that provides precise information regarding the kinetics of gene expression, viral biodistribution, toxicity, and therapeutic outcomes using the accumulation of radiotracers at the site of transgene expression. Here, we provide an overview of pre-clinical and clinical applications of hNIS-based molecular imaging to evaluate virotherapy efficacy. Moreover, we describe different types of reporter genes and their potency in the clinical trials.

## Background

While tumor imaging and radiotherapy strategies are crucial for tumor identification and treatment, the presence of metastasis and radiation resistance has reduced the efficacy of treatment in cancer patients. Consequently, finding novel methods for increasing the efficiency of tumor imaging and treatment is of great importance [1, 2]. Recently, oncolytic virotherapy has provided a new outlook for the therapy of malignancies with promising outcomes [3, 4]. Oncolytic viruses (OVs) specifically induce cancer cells death by infection of and spreading on tumor cells through different mechanisms such as direct lysis effect or adjacent cells fusion. The ability of OVs to selectively infect tumor cells is mainly associated with the disruption of type-I interferon pathway, which represents the first line of defense against viral infections. In this case, the stimulation of endoplasmic reticulum (ER) stress and subsequent immunogenic cell death (ICD) process in OVs-infected cells elicit a range of anti-tumor immune responses. This stress condition can also lead to the attraction of immune cells, notably antigen presenting cells (APCs) (i.e. immature dendritic cells), natural killer cells (NK cells), and cytotoxic T-cells to the site of infection [5, 6].

**Fig. 1:**
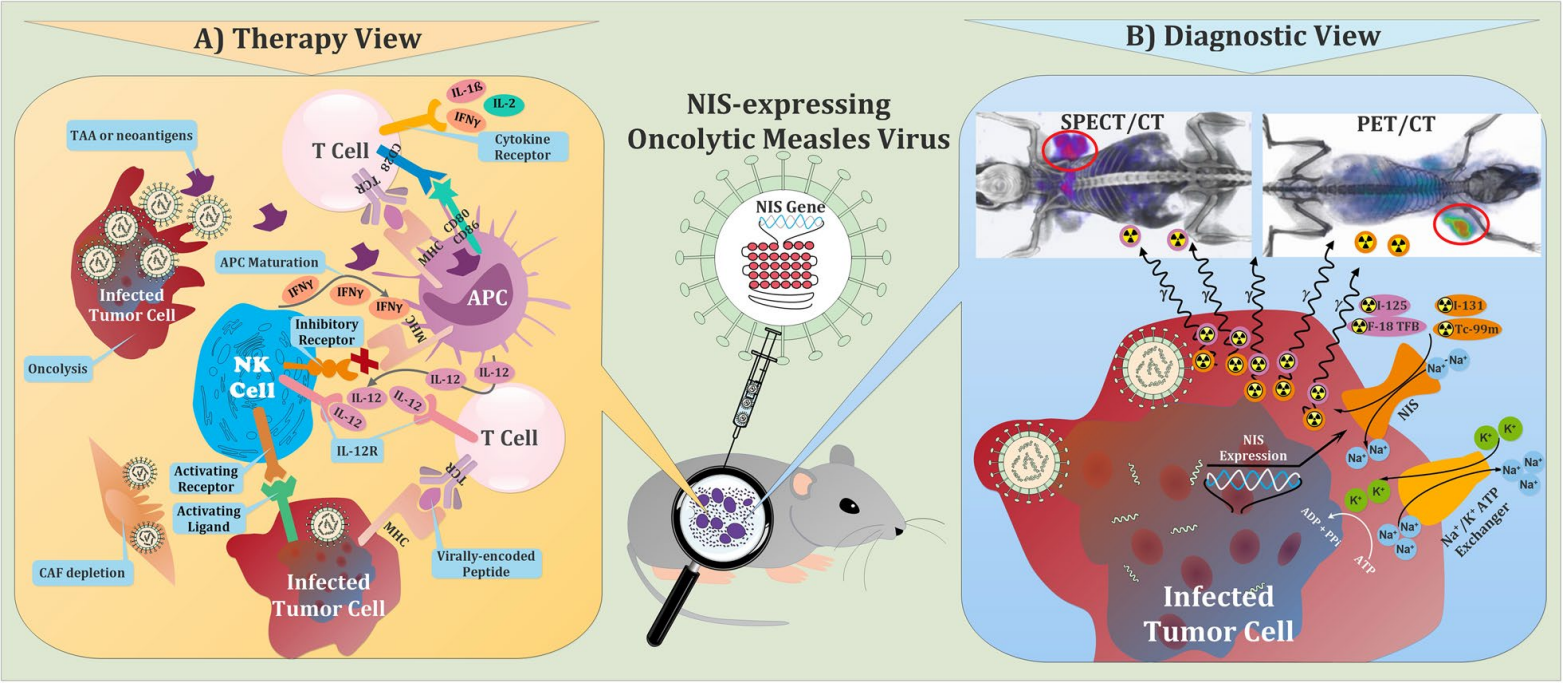
Schematic illustration of the multimodality role of virotheranostics in treatment and tracking of tumor cells. **a** Mechanisms by which oncolytic viruses (OVs) stimulate antitumor immunity. Virus-based immunostimulatory cytokine and chemokine expression can recruit and activate T cells, antigen-presenting cells (APCs), and natural killer (NK) cells, and subsequently, improve the therapeutic activity of OVs. Regardless of oncolysis, OVs stimulate innate immune receptors on professional antigen-presenting cells (APCs) such as dendritic cells and prime antitumor T cells. OVs are able to counteract immune suppression through several mechanisms, including stimulation of pro-inflammatory cytokines and TAAs production and also depletion of immunosuppressive cell types such as cancer-associated fibroblasts (CAFs) within the tumor microenvironment. OVs can also improve recognition of tumor cells by the immune system through upregulation of pathways involved in antigen processing and presentation, including increased major histocompatibility complex (MHC) class I and MHC class II expression on APCs and tumor cells. Subsequently, CD28 signaling will result in the activation of the CD8 + T cell. These activated CD8 + T cells will differentiate into effector T cells that can recognize the MHC class I-peptide complex on virally infected cells. Binding of the TCR to the MHC class I-peptide complex leads to activation of the CD8 + T cell and the release of cytokines. Moreover, NK cells play a pivotal role in detection and killing tumor cells, recruiting other immune cells, mediating T cell activation, and expanding and releasing chemokines and cytokines. The activation of NK cells depends upon the presence of local cytokines such as IL-12. Regulation of NK cells is achieved through binding of inhibitory cell surface receptors such as the killer-cell immunoglobulin receptors (KIRs) (not shown), which bind to different human leukocyte antigen (HLA) complexes on tumor cells. Upon some circumstances of cellular stress like viral infection, associated ligands for activating receptors are often upregulated and MHC class I expression may be downregulated. The upregulation of activating ligands and downregulation of MHC class I produce a signal for NK cells to become activated and play their effector functions. **b** Visualization of tumor cells through NIS-mediated cell imaging. Following transduction with the viral vector carrying the NIS gene, cancer cells are capable of transporting radioisotopes for imaging purposes. Tissue-specific promoter enables the NIS gene to express specifically in corresponding cancer cells, providing a promising strategy of cancer-targeting therapy and imaging. The cells can be imaged by radionuclide-based molecular imaging techniques using gamma-ray or positron-emitting radiotracers. Expression of sodium iodide symporter (NIS) reporter gene leads to the insertion of sodium iodide symporters into the cell membrane, where they import many reporter probes like TcO4^−^, ReO4^−^, and At^−^, along with sodium ion (Na +), into the cytosol. Imaging is performed with SPECT or PET and the results are like example pictures at top of panel B, in which tumor area has been determined by red circle

Upon the stage of tumorigenesis, down-regulation of the major histocompatibility complex class-I (MHC-I) molecules and also the overexpression of the stress-induced ligands on the surface of malignant cells to avoid recognition by cytotoxic T-lymphocytes (CTLs), make them more sensitive to NK cell-mediated death. In this manner, the reduced detection of self MHC-I molecules by NK cell-inhibitory receptors coupled with an increase in activating signals by recognition of stress-induced surface ligands promote the cytolytic activity of NK cells. Of note, perforin and granzyme released by activated NK cells represent major lytic mediators involved in the destruction of tumor cells [7, 8]. On the other hand, the proliferation of oncolytic viruses into target cells and subsequent recognition of viral elements by pattern recognition receptors (PRRs) such as toll-like receptors can positively affect the development of the innate and adaptive immune responses against tumor cells (Fig. 1) [6].

To perceive and prognosticate therapeutic outcomes of therapy with OVs, various mathematical models have been expanded. The improvement of such models, which are capable of accurately measuring in vivo dynamic proliferation, is accessible by means of technologies that allow quantitative real-time measurements in tumor cells. Accordingly, engineered OVs have attracted more attention in accurate tumor imaging and treatment because they are capable of expressing reporter genes and demonstrating treatment efficacy in addition to killing cancer cells [9, 10]. Oncolytic measles virus (OMV) as a derivative of the Edmonston vaccine strain is an RNA virus belonging to the *Paramyxoviridae* family, which its employment safety has been confirmed in many human malignancies [11, 12]. The OMV demonstrates a tropism for the CD46 membrane protein, part of the complementary regulatory pathway, which is expressed at high levels on tumors compared to normal cells. Infection of tumor cells by OMV induces cell death via apoptosis and syncytia formation [13].

Many preclinical studies have shown high efficacy of OMV vectors in melanoma, ovarian, and hepatocellular and squamous cell carcinoma models. Measles virus expressing the sodium iodide symporter (NIS) is an Edmonston vaccine strain of MV, engineered to express the NIS as the most prevalent imaging reporter gene. The NIS gene expression by OMV enables noninvasive monitoring of cancer cells and enhances anti-tumor response through the uptake of radiolabeled iodine [14]. In this review, the use of NIS-based molecular system to combine virotherapy and molecular imaging areas in pre-clinical and clinical settings, as well as the ability to use different types of reporter genes in the clinical trials will be studied.

### Obstacle and approaches for improving virotherapy

Two basic steps to increase the efficacy of oncolytic virotherapy are reaching the appropriate amount of virus at the tumor site and the spreading ability of the virus within the tumor tissue. Depending on the administration route of oncolytic viruses to cancer therapy, many barriers such as trapping virus particles by liver, spleen, or neutralizing antibodies, and activation of the immune responses against viral replication can hamper virus delivery to target organ and greatly impress the effectiveness of treatment [15, 16]. The adaptive immune system has the most significant negative effect on treatment efficacy, which can affect both delivery and spread of the OMV in tumor tissue. Moreover, the presence of pre-existing antibodies against the measles virus reduces the efficacy of therapy in the intravenous route, especially in repeated systemic administration. However, different solutions have been suggested to reduce these effects [17]. One of these strategies is the administration of cyclophosphamide (CPA) as an anticancer and suppressor agent to prevent tissue rejection in transplanted patients. CPA works by killing disturbing lymphocytes such as T, B, and NK cells. One study showed that the administration of CPA 1 week before OMV therapy increased oncolytic efficacy through short-term immunosuppression in tumor tissue [18, 19].

Another strategy to avoid neutralizing antibodies is the use of carrier cells. Various cells such as mesenchymal stem cells (MSCs), dendritic cells, and activated T cells can be used for this purpose. Due to their exceptional features such as homing to tumor tissues as well as immunomodulatory and self-renewal capacity, MSCs have received special attention and can be used for metastatic cancers [20, 21]. Other strategies, such as the covering of oncolytic virus particles with nanoparticles and the construction of chimeric particles are also effective in reducing the malfunction of the immune system. A study by Mader et al. revealed that despite the presence of neutralizing antibodies, MSC-loaded OMV induced syncytia formation in an orthotopic ovarian cancer model and increased survival compared to naked OMV or uninfected MSC groups [20].

### Overall-molecular imaging

Molecular imaging is a powerful tool that enables clinical studies to noninvasively and serially detect viral targeting sites. Additionally, the viral infection level is also measurable through molecular imaging, which can provide information on the toxicity, efficacy, and safety profile of oncolytic viruses [22, 23]. Generally, real-time tracking of virus evolution can optimize treatment by providing useful information regarding the viral dose and administration schedule, as well as the elimination of the need for multiple and repeated tissue biopsies. Moreover, the ability of molecular imaging to improve vector design and clinical protocols facilitates the use of personalized medicine in the treatment of cancer [22, 24–26].

There are various imaging modalities for molecular imaging and based on the employed molecular probe are divided into two groups including non-nuclear and nuclear molecular imaging modalities. Bioluminescence and fluorescence imaging devices (e.g. charge-coupled device (CCD) cameras) are the most common imaging modalities applied in non-nuclear medicine. These external imaging devices use light as a nonradioactive probe and are predominantly restricted to preclinical assays [25, 27]. Nevertheless, nuclear molecular imaging tools such as SPECT, positron emission tomography (PET) scanner, and gamma camera modalities can potentially be used in clinical trials and due to their ability to recognize and localize the gamma-ray emitted by a radioactive tracer decay, they can provide more reliable information about virotherapy outcomes. These scanners are also very sensitive and yield better spatial resolution [9, 27]. To improve the spatial resolution and provide more information concerning the anatomy of target tissues and the level of gene expression, metabolism, and receptor distribution, hybrid imaging systems using the combination of visualization instruments such as PET and SPECT with computed tomography (CT) or magnetic resonance imaging (MRI) have been developed [28].

Of note, serial monitoring of infection, prediction of the primary site of tumors and metastases, as well as evaluation of antitumor efficacy are possible using the genetically engineered viruses that express a reporter gene, which can be imaged by molecular imaging modalities [22, 25, 28]. In this manner, the accumulation of tracer molecules at the site of transgene expression and recognition of their emitted signals by external imaging devices is used to follow the viral evolution in tissues of interest. It is worth noting that these targeting moieties or signaling agents introduced as imaging molecular probes should not be immunogenic, or otherwise interfere with the function of subject cells [25, 27, 29].

**Fig. 2:**
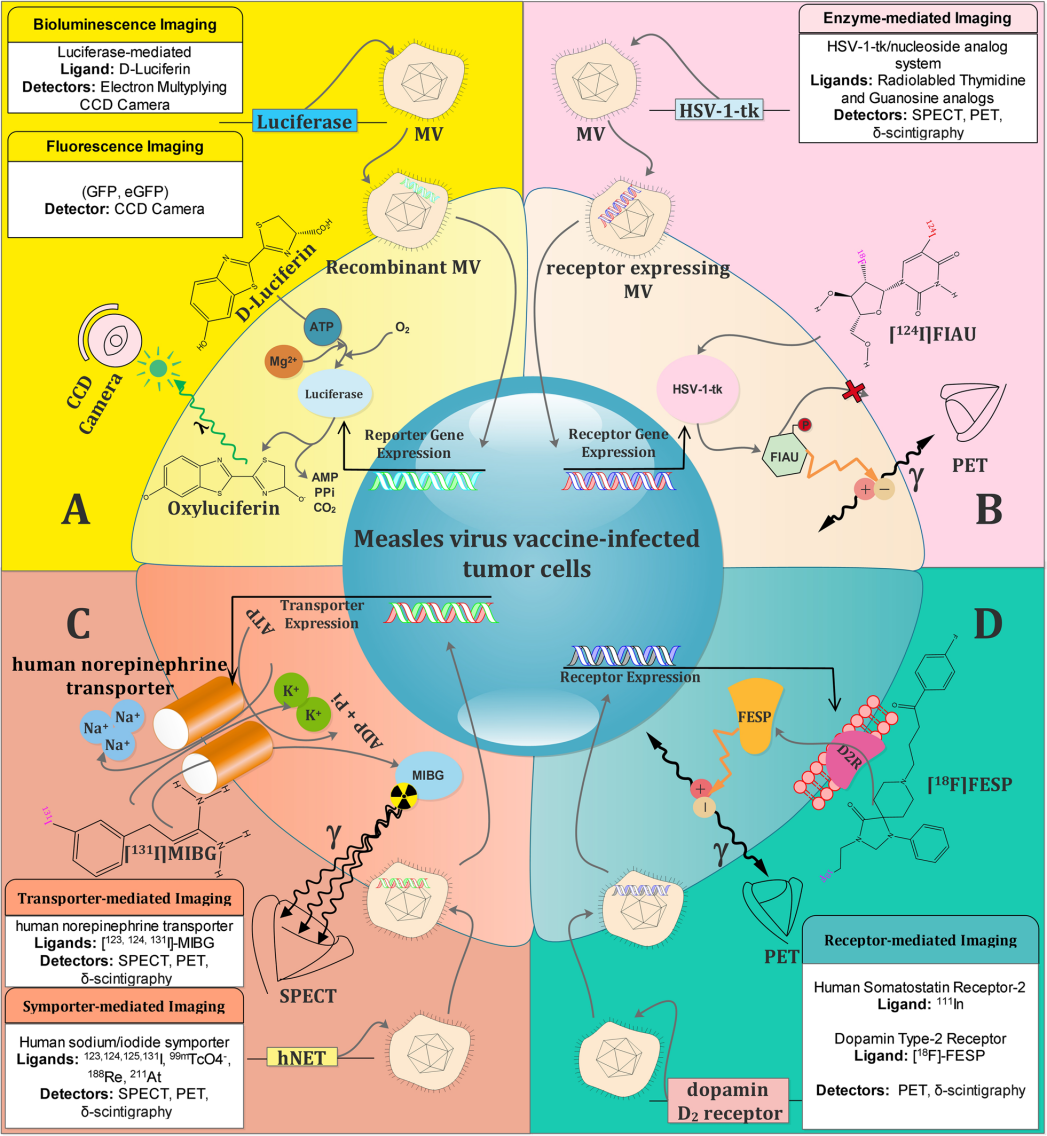
Schematic illustration of tumor cell imaging using noninvasive visualization systems by means of measles viruses (MVs) to encode reporter transgenes based on recombinant technology. **a** Optical imaging. Transcription and translation of the firefly luciferase (FLUC) gene lead to the accumulation of the firefly luciferase enzyme, which subsequently catalyzes a photochemical reaction in the presence of D-luciferin. This reaction yields low levels of fluorescence photons that can be detected and quantified by a charge-coupled device camera (CCD). **b** Enzyme-mediated imaging. Inside the transfected cells, HSV-1-tk is transcribed and translated to produce the HSV-1-tk enzyme. [^124^I]-FIAU is a labeled nucleoside analog substrate for HSV-1-tk. In the presence of HSV-1-tk, the radiolabeled probe is phosphorylated and trapped within the cell. Radioactive decay of ^124^I isotopes can be detected with PET. The magnitude of [^124^I]-FIAU signal reflects the activity of HSV-1-tk enzyme and thus HSV-1-tk gene expression. **c** Transporter/Symporter-mediated imaging. Tumor cells would acquire the function of iodine uptake with NIS gene transduction by viral vector delivery. NIS transports 2 sodium ions and 1 iodide ion into the cytoplasm together. The electrochemical sodium gradient generated by the Na^+^/K^+^ ATPase pump provides energy for this transfer. Gamma radiation of radiolabeled ligands such as [^131^I]-MIBG provide enough radiation for PET imaging. **d** Receptor-mediated imaging. 3-(2′-[^18^F]fluoroethyl)spiperone (FESP) is a gamma-emitting radiolabeled reporter probe that interacts with the dopamine 2 receptor (D2R) to result in probe trapping on or in cells expressing the D2R gene. Viral delivery of the D2R gene into the infected tumor cells armors them with a monitoring system, which can be visualized by PET

Regarding the use of radioactive and non-radioactive substrates as targeting moieties, molecular imaging methods are broadly categorized into optical and deep-tissue imaging systems [25]. In the following sections, we will focus on the most investigated reporter transgenes based on the type of imaging technique (Table 1). The mechanism of molecular imaging methods based on reporter genes is shown in Fig. 2.

### Optical imaging

Optical detection techniques are introduced as a promising approach to evaluate oncolytic viruses’ activity in small living subjects using non-nuclear molecular imaging modalities and include fluorescence and bioluminescence imaging [25, 30]. Due to its radiation safety profile, quick performance, and low cost, optical imaging is considered as the main non-invasive visualization method in preclinical assays. However, the application of these techniques for deep-tissue imaging has been limited by light scattering in biological tissues, auto-fluorescence emitted by dead cells, and presence of cellular inhibitors of excitation below 600 nm [25].

### Fluorescence-based reporter transgenes

Fluorescent proteins are broadly used for in vivo imaging of viral gene expression in small tumor-bearing animals. Jellyfish *Aequorea Victoria*-derived green fluorescence protein (GFP) is the most prevalent optical reporter transgene cloned into the oncolytic viral genome. However, due to the limited penetration depth of light required for GFP excitation and emission, this reporter protein commonly used for following the colonization of OVs in tumors and metastases close to the body surface [25, 29]. In order to optimize the brightness and increase the structural stability of GFP without disturbing its function as a reporter protein, mutation screen has run and among the recombinant constructs, a human codon-optimized variant of GFP, enhanced GFP (eGFP, F64L/S65T), has shown prominent results in molecular imaging assays [31]. Better packing of the eGFP due to geometrical properties of L64 in comparison with F64 in GFP improves the stability of protein and adds extra capacity for inter-residual communication, resulting in improved function in different contexts [32].

### Luciferase-based reporter transgenes

Luciferase-mediated bioluminescence imaging (BLI) has been initially used to follow the evolution of spreading infection within target tissues in small living animals. In this system, the intensity of emitted light depends on the enzyme efficacy in catalyzing the bioluminescence reaction and the concentration of substrate and cofactor (ATP-Mg^2+^) [25, 33]. Compared with fluorescence, the luminescence system is more sensitive due to the lower background noise levels. Therefore, this approach is more applicable than fluorescence for whole-body imaging and the prediction of the primary site of near-surface tumors and metastases. However, given the low spatial resolution, BLI is not available for large animals and is limited to experimental models [25, 29]. Due to the small size, the luciferase substrate (luciferin for firefly luciferase and coelenterazine for Renilla and Gaussia luciferases) easily crosses the plasma membrane and reaches to the target tissues; thereafter, the visible light emitted upon enzymatic oxidation of the substrate at the site of luciferase expression enables virus tracking [29, 33].

### Deep-tissue imaging

To address several limitations of optical imaging including restricted application to small models, difficulties in quantitation, and limited depth penetration of a few millimeters, the design of a more sophisticated molecular imaging technique is required [34]. Currently, based on enzymatic activity, specific receptors, and ion transporter/symporter, three classes of reporter transgene for use in deep-tissue imaging are developed via nuclear medicine techniques [29]. We introduce these types of transgenes in the following sections.

### Enzyme-based reporter transgenes

Herpes simplex virus-1 (HSV-1) thymidine kinase (TK) gene is the most studied reporter transgene based on enzymatic activity. Oncolytic virus-mediated tumor eradication can be evaluated noninvasively using the phosphorylation of radiolabeled thymidine analogs such as [^125^I or ^124^I]-FIAU (fial2′-fluoro-2′-deoxy-1-β-d-arabinofuranosyl-5-iodouracil), as well as radiolabeled guanosine analogs like 8-[^18^F]-fluoroganciclovir (^18^F-FGCV) and [^18^F]-FHBG (9-(4-fluoro-3-hydroxymethylbutyl) guanine) by HSV-1-TK [25, 35]. After the phosphorylation of cell-permeable radiotracers, the negative charge of the phosphate group (polar form) traps them into the cytoplasm of cancer cells. Accordingly, the accumulation of trapped radiotracers can be used as an indicator for in vivo monitoring of viral oncolysis activity over time [36, 37]. The main advantage of the HSV-1-TK/nucleoside analog system is its bystander-killing effect by transferring phosphorylated nucleoside analogs from infected cells to surrounding un-transduced tumor cells using the gap junctions or apoptotic vesicles, which enhances the efficacy of virotherapy [36, 38]. However, some disadvantages are attributed to this system including (a) limited clinical usage due to the regulatory restrictions associated with the radiolabeled thymidine analogs as investigational drugs, and (b) increased undesirable immune responses related to the non-human origin of exogenous reporter gene [25].

### Receptor-based reporter transgenes

Specific receptors such as human somatostatin receptor 2 (hSSRT2) and dopamine type-2 receptor (D2R) can be employed in serial monitoring of oncolytic viruses delivery into malignant cells through binding and trapping the radiolabeled ligands [29]. The hSSRT2 is typically found in human kidney cells, as well as in neuroendocrine tumors and is targeted by the high-affinity synthetic peptide pentetreotide. Regarding the ability of systematic administration of hSSRT2-expressing oncolytic viruses, intratumoral delivery of hSSRT2 allows tracking of all primary and metastatic tumor sites [39]. However, an important disadvantage of this reporter system is the need for prior radiolabeling of tracer molecules. Alternatively, given the binding ratio of 1:1 between the receptor and its corresponding radiotracers, the sufficient signal for viral monitoring may not be produced during in vivo imaging [25]. The D2R is another human receptor that is exclusively expressed in the striatum and pituitary and is targeted by high-affinity radiolabeled ligands like 3-(2′-[^18^F]fluoroethyl)spiperone (FESP). Therefore, in this visualization system, the use of exogenous receptors do not elicit undesirable immune responses [40]. However, given the high lipophilicity of D2R ligands, the accumulation and excretion of validated D2R ligands in liver cells upon systemic administration are inevitable [41].

### Transporter-based reporter transgenes

Human norepinephrine transporter (hNET) is a Na^+^/Cl^−^-dependent cell-membrane transporter protein involved in the movement of norepinephrine, dopamine, and epinephrine through the plasma membrane [25]. Although the expression of hNET is predominantly restricted to the central and peripheral sympathetic nervous systems, it is also found at high levels in neuroendocrine tumors and is targeted by the clinically approved metaiodobenzylguanidine (MIBG) [25, 42]. Regarding some advantages attributed to hNET such as (a) the human origin of hNET and its local expression in normal tissues, and (b) the ability to radiolabel the MIBG with gamma-emitting radioisotopes of iodine (^123^I, ^124^I, and ^131^I), which results in serial imaging through SPECT and γ-camera imaging (^123^I, and ^131^I), as well as PET system (^124^I), hNET can be employed as a potential human reporter gene for noninvasive imaging [25, 42].

### Symporter-base reporter transgene

Human sodium/iodide symporter (hNIS), a prevalent nuclear medicine reporter gene, is an integral glycoprotein of the basolateral cell membrane, enabling the thyroid gland follicular cells to regulate intracellular iodide trapping [39, 43]. This process is started by the NIS-mediated co-transport of two sodium ions along with one iodide ion into thyrocytes. The required energy for this co-transport against the cellular electrochemical gradient is provided by the sodium/potassium (Na^+^/K^+^) ATPase [43]. Following iodide (I^−^) uptake, molecular iodine (I_2_) is produced by the oxidation of iodide using thyroid peroxidase enzyme (TPO). Iodine is one of the important constituents of thyroid hormones (THs), which plays a pivotal role in THs synthesis [27, 44]. In this way, upon the process called iodide organification, oxidized iodide is covalently coupled to thyroglobulin (Tg), a protein found in the follicular lumen at high levels that serves as the precursor of thyroid hormones in iodinated form and also constitutes the storage form of iodine in the thyroid gland [27].

Besides iodide uptake, intracellular accumulation of anionic substrates such as pertechnetate (TcO_4_^−^), perrhenate (ReO_4_^−^), and astatide (At^−^) can also be promoted by hNIS-expressing cells. Human sodium/iodide symporter is distributed exclusively in thyroid tissues and to a lesser extent in some extra-thyroidal tissues including salivary glands, gastric mucosa, and lactating mammary glands [44]. Since iodide organification is a unique feature of the thyroid cells, the ability to retain iodide in extra-thyroidal tissues is reduced. The exact physiological role of NIS transporter in extra-thyroidal tissues has not been clarified until now [27]. Experimental observation also has shown that the delivery of the hNIS gene to non-thyroid tumor cells would trigger iodide uptake [44]. These findings suggest that ectopic hNIS expression in tissues of interest could play a significant role in the real-time noninvasive molecular imaging using the intracellular accumulation of anionic radiotracers such as gamma-emitting radioisotopes of iodide (^123^I, ^124^I, ^125^I, and ^131^I), ^99m^TcO_4_^−^, ^188^Re, and ^211^At at the site of gene expression [25].

Compared to other reporter genes, the use of NIS has several potential advantages including (a) the human-origin of NIS and its clinically approved radiotracers; (b) no need for prior radiolabeling of iodide due to its radiotracer property, which reduces corresponding costs; (c) the ability to use various imaging systems such as PET, SPECT, and γ-camera imaging to monitor viral distribution due to the cellular uptake of various radioisotopes by NIS [25, 27]; (d) the provision of anatomical information of NIS-expressing infected cells by the combination of SPECT or PET with CT [22]; (e) the ability to reflect the cell viability due to lost NIS concentrative function upon cell apoptosis [22, 45]; (f) improvement in sensitivity of detection due to the signal amplification through the transport-mediated concentrative intracellular accumulation of substrate [46]; and (g) the ability to concentrate carrier-free radiotracers for convenient use [22].

Moreover, given the extensive emission range of β particles produced by ^131^I (0.4 mm), the likelihood of killing non-transduced malignant cells increases by irradiating with radiation emitted from adjacent infected cells. This effect, termed the “physical crossfire”, can potentially be used in radiovirotherapy. However, it may also result in unwanted toxicity through damage to neighboring healthy cells [27, 47]. It is worth noting that combination of NIS-mediated imaging with ionizing radiation (e.g. ^131^I) may have a synergistic effect in cancer therapy, which can be attributed to viral replication increase in response to upregulation of some DNA repairing pathways or also α decay particles produced by ^131^I (0.2–2.4 mm in path length) [48, 49].

One of the disadvantages of this reporter system is its inability to organify iodide in extra-thyroidal tissues, which reduces the intracellular retention capacity of radiotracers in NIS-transduced cells. In this regard, after intracellular uptake, efflux pump is employed to export the radiotracers out of the cell, which may interfere with in vivo imaging. Nevertheless, it has been determined that radiotracers’ efflux occurs at slow rate. Thus, it seems that the residual of radiolabeled iodide is enough to generate the required signal for invasive in vivo imaging. Use of alternative strategies such as employing radiotracers with greater energy (e.g. ^188^Re) to enhance local signal or co-delivery of NIS and TPO to help iodide organification in non-thyroidal tumor cells can be useful to overcome this limitation [27, 44].

Another limitation of serial hNIS imaging is the probability of radiotracers uptake by both malignant and normal tissues due to the extensive distribution of hNIS protein in normal tissues, which can lead to reduced radiotracers accumulation into targets of interest [50]. Furthermore, iodide shows a complex and unfavorable biodistribution and a large percentage of radioiodide is accumulated in the thyroid (up to 25% of the injected dose in euthyroid patients). Pretreatment with thyroid hormones for several days will be required to reduce this uptake. Additionally, the administration of contrast media for radiological studies would significantly reduce radioiodide uptake by NIS-transduced cells due to the competition between radioiodide and stable iodide released from the contrast media (Table 1) [51, 52].

**Table 1:** Oncolytic viruses encoding reporter transgenes for various imaging modalities

Visualization methods	Reporter transgenes		Oncolytic viruses encoding reporter transgenes	Imaging modalities	Ref.
Optical imaging	Fluorescent proteins (e.g., GFP, eGFP)		Newcastle disease virus, measles virus, herpes simplex virus-1 (HSV-1), adenovirus, vaccinia virus (GLV-1h68), and Vesicular stomatitis virus (VSV)	Specially adapted CCD cameras to detect fluorescence	[25, 29, 33]
	Luciferase		Parvoviruses, adenoviruses, HSV-1, vaccinia virus, measles virus, and VSV	EM-CCD camera	
Deep-tissue imaging	Enzyme-mediated imaging (e.g., HSV-1-TK)		Sindbis virus	SPECT and PET scanner, and γ-scintigraphy	[27, 29, 36]
	Receptor-mediated imaging	hSSRT2	Vaccinia virus	PET scanner and γ- scintigraphy	[25, 27, 34, 40]
		D2R	Adenovirus	PET scanner	
	Transporter/Symporter-mediated imaging	h-NET	Vaccinia virus (GLV-1h99)	SPECT and PET scanner, and γ-scintigraphy	[42]
		h-NIS	Adenoviruses, measles, vesicular stomatitis, vaccinia virus (GLV-1h153), HSV, and Retrovirus	SPECT and PET scanner, and γ-scintigraphy	[25, 27, 44]

*CCD camera* Charge-Coupled Device, *EMCCD Camera* Electron multiplying CCD camera, *SPECT* single-photon emission computed tomography, *PET* positron emission tomography

### Tumor imaging using oncolytic measles virus

According to several investigations, oncolytic virus therapy is a novel frontier for clinical trials [53]. The major purpose of replication-competent cancer virotherapy is to produce a safe and efficient therapeutic index with minimal toxicity [25, 54]. To date, many studies have revealed that attenuated measles virus of the Edmonston lineage (MV-Edm) has antitumor activity in several types of cancer and also is able to minimize the damages in normal cells as stated earlier [22, 55–60]. Recently, owing to the strong oncolytic potential of MV-Edm and the above-mentioned advantages of NIS as a reporter gene, several groups have developed an engineered, attenuated measles virus expressing the NIS gene (MV-NIS) to evaluate the feasibility of NIS reporter gene for noninvasive imaging and monitoring of MV-NIS delivery. Table 2 lists preclinical and clinical studies in which MV infection has been detected noninvasively using molecular imaging techniques, and also shows the mechanism of imaging of oncolytic MV by image-guided injection techniques.

**Table 2:** Preclinical and clinical studies on in vivo monitoring and imaging of measles virus (MV) infection, replication, and expression

Study/year	Imaging technique	Tumor type	Data sources	Genes/protein	Route	Protein targeting drug	Main results
Deyle et al./2015 [55]	SPECT/CT	Malignant peripheral nerve sheath	Athymic nude mice	hNIS	IT	^125^I	MV localization and distribution could be monitored by imaging of I-125 uptake
Dingli et al./2004 [56]	Gamma camera	Myeloma	Mice	hNIS	IV	^123^I	In vivo replication of MV-NIS peaked 9 days after virus injection
Galanis et l./2015 [58]	SPECT/CT	Ovarian cancer	Human	hNIS	IP	^123^I	No dose-limiting toxicity was observed in 16 patients treated at the 10^8^–10^9^ TCID_50_ dose level; all observed toxicities were grade 1 and 2
Hasegawa et al./2006 [75]	Gamma camera and bioluminescence	Ovarian cancer	Mice	hNIS; blood CEA	IT, IV	Tc-99 m sodium pertechnetate; luciferase (Fluc) and bhCG	Viral gene expression was monitored by measuring blood CEA levels, and the location of virus-infected cells was monitored by gamma camera imaging; The gamma camera scans were significantly less sensitive than the plasma CEA marker for monitoring virus infection
Hutzen et al./2012 [65]	Bioluminescent (Cherenkov) imaging	Medulloblastoma	Mice	hNIS	IT	D-Luciferin	The MV-NIS mouse indicated an increased bioluminescent signal originating from the tumor that ^131^I had accumulated
Msaouel et al./2009 [64]	Gamma camera	Prostate	Nude mice	hNIS	IT, IV	^123^I	In vivo replication of MV-NIS depends on the administration route. Strong positive tumor ^123^I uptake is seen 4 days after IT administration of MV-NIS, and 14 days after IV administration of MV-NIS. Persistent transgene expression can be detected for as long as 36 days after IV administration of the virus
Myers et al./2007 [19]	–	Myeloma	SCID mice, squirrel monkey	hNIS	IV	^123^I	No adverse effect was observed
Opyrchal et al./2012 [67]	Gamma camera	Glioblastoma	BALB/c nude mice	hNIS	IT	^123^I; Tc-99 m sodium pertechnetate	Tumor uptake of radioisotope in MV-NIS-treated mice was increased; peak at day 3 and persistence at 20 days after viral administration. Expression of NIS protein in infected cells results in the effective concentration of radioactive iodine that allows for in vivo monitoring of localization of MV-NIS infection by measuring uptake of I-123 and Tc-99 m
Penheiter et al./2012 [62]	SPECT/CT	Pancreatic	Nude mice	hNIS	IT, IV	^123^I	Pinhole micro-SPECT/CT imaging using the NIS reporter allows for precise localization and quantitation of oncolytic MV-NIS infection. This method can replace autoradiography and Immunohistochemistry analysis
Penheiter et al./2012 [77]	SPECT/CT	Pancreatic	Nude mice	hNIS	IT	Tc-99 m sodium pertechnetate	IT viral delivery can be monitored using image-guided injection techniques
Penheiter et al./2010 [59]	SPECT/CT	Pancreatic	Nude mice	hNIS	IT	^123^I	Delivery of ^131^I radiotherapy to NIS-expressing tumors can be optimized using micro-SPECT/CT imaging guidance. In vivo viral replication was variable among mice (peak between 2 days and 6 days following viral administration, and undetectable at 9 days after viral injection)
Reddi et al./2012 [73]	SPECT/CT	Anaplastic thyroid	Nude mice	hNIS	IT	Tc-99 m sodium pertechnetate	NIS expression peaked at day 3 and was persistence up to day 22 following viral administration
Russell et al./2014 [60]	SPECT/CT	Myeloma and plasmacytoma	Human	hNIS	IV	^123^I	In all the two patients, tumor imaging was clearly documented. The size of the lesions was variable in hNIS mediated radioiodine SPECT-CT for the same lesions shown in the imaging in comparison with the normal FDG PET-CT. In vivo viral replication depends on patient and plasmacytoma. Radioiodine uptake returned to the background at day 28 following MV-NIS administration
Carlson et al./2009 [61]	Gamma camera, SPECT/CT	Pancreatic	Nude mice	hNIS	IT	^123^I	Mice infected with MV-NIS concentrated radioiodine, that allows for serial quantitative imaging with ^123^I micro-SPECT/CT. The peak iodide uptake was 2 days after MV-NIS administration
Dispenzieri et al./2017 [57]	SPECT/CT	Multiple myeloma	Human	hNIS	IV	^123^I	8 out of the 31 patients had some degree of ^123^I uptake on their SPECT/CT scans
Miest et al./2013 [72]	SPECT/CT	Mantle cell lymphoma	SCID mice	hNIS	IT, IV	Tc-99 m sodium pertechnetate	NIS gene results in concentrating iodide within infected cells, allowing non-invasive imaging. High-resolution imaging visualized the spread of infections in primary and metastatic tumors for over 2 weeks after treatment, documenting homogeneous virus seeding and spread restricted to perfused tissue
Jung et al./2018 [78]	SPECT/CT	Pancreatic	Nude mice	hNIS	IT	Tc-99 m	In vivo radioisotope uptake was significantly correlated with viral N and NIS gene expression
Kemler et al./2019 [63]	Intravital imaging	Fibrosarcoma	Athymic nude mice	Blue fluorescent protein containing the nuclear localization sequence	–	–	Intravital imaging system using the dorsal skin fold chamber allows for serial, non-invasive imaging of tumor cells and replication of a fusogenic and a hypofusogenic MV. There were distinctly different replication kinetics and phenotypes of these two viruses
Li et al./2012 [66]	SPECT/CT	Squamous cell carcinoma	Athymic nude mice	hNIS	IT	^125^I	In vivo viral replication peak occurred 3 days after viral administration

*SPECT/CT* Single-photon emission computed tomography/computed tomography, *hNIS* Human sodium iodide symporter, *IT* Intra-tumoral, *IV* Intravenous, *IP* Intraperitoneal, *MV* Measles virus, *PET* Positron emission tomography

Employing a recombinant MV-Edm expressing the hNIS, Dingli et al. showed that intratumoral spread of MV-NIS can be monitored noninvasively in vivo using serial gamma-camera imaging of ^123^I. In this study, NIS expression and ^123^I uptake were observed 9 days following intravenous injection of MV-NIS into mice. The results of serial imaging after a single injection of MV-NIS demonstrated changes in iodide uptake owing to viral and tumor replication [56]. In another study, Carlson et al. have evaluated the use of NIS for monitoring and quantitation of MV-NIS delivery, viral spread, and gene expression in pancreatic cancer [61]. Due to the close anatomical proximity of pancreas and stomach, uptake of radioiodine in the stomach can produce strong signals that may overlap with pancreas signals and result in reducing spatial resolution. To resolve this problem and improve three-dimensional spatial resolution, the involvement of cross-sectional fusion imaging techniques such as SPECT/CT and PET/CT is necessary. In this regard, planar and micro SPECT/CT images indicate tumor xenografts infected with MV-NIS concentrated radioiodine in vivo, providing serial noninvasive imaging and quantitation of radionuclide uptake [61].

In addition to the detection of heterogeneity in replication kinetics and biodistribution of infection, exploring the origin of heterogeneity is possible through the NIS-mediated noninvasive imaging and monitoring [22]. A recent study by Penheiter et al. has successfully employed pinhole micro SPECT/CT imaging to resolve intratumoral viral dispersion patterns in a human pancreatic xenograft model infected with MV-NIS [62]. The results suggest that the use of micro SPECT/CT for localization and quantification of the oncolytic MV-NIS infection is less time-consuming and more cost-effective in comparison with autoradiography and immunohistochemical analysis that require animal killing [62]. In another study by the same group, the role of micro SPECT/CT in optimizing the timing of MV-NIS-induced oncolysis and NIS-mediated ^131^I radiotherapy of human pancreatic cancer xenografts in athymic nude mice was determined. Serial imaging with ^123^I micro-SPECT/CT after virus injection revealed significant temporal variability in peak tumor iodide localization and tumor reaction after intratumoral MV-NIS injection [59].

Deyle et al. were the first to show the feasibility of noninvasive theragnostic potential of MV-NIS for malignant peripheral nerve sheath tumor (MPNST). In vivo monitoring of MV-NIS using ^125^I SPECT/CT imaging showed that radioiodine uptake in tumors infected with MV-NIS was approximately 2.5 times higher than control tumors. Consequently, MV-NIS propagation in MPNST tumors can be monitored noninvasively in vivo using ^125^I SPECT/CT [55]. Galanis et al. used NIS-mediated imaging to clinically monitor MV-NIS delivery in resistant ovarian cancer patients after intraperitoneal MV-NIS administration every 4 weeks for up to 6 cycles. NIS expression was imaged by ^123^I uptake on SPECT/CT and in 3 out of 13 patients treated at the 10^9^ TCID_50_ dose level, NIS expression was observed. The results showed that there is a temporal heterogeneity in the detection of NIS-mediated radiotracer uptake, confirming differences in intratumoral virus replication kinetics or initial intratumoral deposition of virus particles [58]. The results of a phase I trial showed that MV encoding NIS can be a treatment option for refractory multiple myeloma [57]. Russell et al. presented imaging data and clinical response of two measles-seronegative myeloma patients treated with intravenous infusion of 10^11^ TCID_50_ MV expressing NIS. Their results showed that uptake intensity is different among tumors of similar size, which proves the heterogeneity in viral replication even within the same patient. Furthermore, in both cases, the treatment resulted in M protein reduction and bone marrow plasmacytosis resolution [60].

Of note, the most important limitation of some imaging techniques such as micro SPECT/CT and bioluminescence is restricted spatial resolution (0.25 mm and 1–2 mm, respectively) [63]. Alternatively, they provide information on the macroscopic scale. In an attempt to image the replication of an MV in vivo in tumors over a time span of several days at single-cell resolution, Kemler et al. have recently established an intravital imaging system using the dorsal skinfold chamber (DSFC) and two-photon microscopy. In this study, they compared the replication and oncolysis of a fusogenic and a hypofusogenic MV. The results showed distinctly different replication kinetics and phenotypes of these viruses at cellular level [63]. Preclinical and clinical studies have demonstrated the ability of NIS-mediated imaging of radiotracer uptake to noninvasively monitor OMV replication and propagation. The results of ongoing clinical trials with MV expressing NIS show that NIS-mediated imaging can provide information about pharmacokinetics and treatment response for clinicians, but is in need of further investigation for future clinical applications.

### NIS-mediated therapy

During the last two decades, different malignancies with various origins are under treatment by MV-NIS and the acquired results are highly promising. An early study on radiovirotherapy of myeloma tumors in mice represented complete regression of MV-sensitive tumors after MV-NIS administration and also regression of MV-resistant tumors after the addition of ^131^I [56]. Msaouel et al. showed that the administration of a single dose of MV-NIS to prostate cancer xenografts in mice could significantly increase tumor regression and prolong mice survival. Furthermore, subsequent administration of ^131^I significantly enhanced the effectiveness of the method and induced tumor eradication in 40–60% of cases. Interestingly, increasing doses up to sixfold resulted in the complete elimination of tumors in 80% of mice models [64]. Similarly, in another survey, it has been demonstrated that there is a correlation between the injection number and a reduction in tumor growth [61].

Other studies also have shown the lethal impact of MV-NIS on various cancer types. While utilization of both MV-NIS alone or in combination with ^131^I resulted in the destruction of medulloblastoma cells in vitro and regression of mouse medulloblastoma xenografts, administration of ^131^I yielded a stronger response [65]. Similar results were observed for SCCHN [66] and glioblastoma [67] in vitro and in vivo and an effective concentration of radioiodine ^131^I in the tumors following administration of MV-NIS was evident in both cases. The administration of this oncolytic agent mediates antitumor effect through inducing severe cytopathic effects (CPEs). Msaouel et al. Observed significant CPEs following the addition of MV-NIS to prostate cancer cell lines [64]. The same CPEs were reported by Carlson et al. where syncytia formation and malignant cell death occurred after in vitro infection of human pancreatic cancer cell lines with MV-NIS [61]. Additionally, another study showed that MV-NIS administration induces considerable CPEs in MPNST cell lines [55]. In addition to their highly effective therapeutic role, these particles have acceptable safety and tolerability [64]. In a clinical survey on ovarian cancer patients, dose-limiting toxicity did not occur even at high doses of MV-NIS [69] and also Russell et al. have reported that the mild toxicity of the MV-NIS-based therapy approach was eliminated within a week [60].

Many cancer types like prostate tumors have a strong expression level of measles receptor CD46 [68–71], which facilitates effective infection of tumor cells by this oncolytic virus. A study showed that higher expression of CD46 on myeloma tumors compared to healthy tissues mediates tropism of the virus for malignant plasma cells and higher sensitivity of cancer cells to infection and destruction by MV-NIS [56]. Similarly, administration of MV-NIS into MPNST cell lines with the high expression level of the CD46 receptor on their surface resulted in severe CPEs, whereas normal cells suffered much less damage [55]. Due to this higher permissiveness of malignant cells for MV-NIS, not only MV-NIS-based therapies in combination with systemic ^131^I result in a better therapeutic response in primary tumors than healthy tissue, but also mediate higher isotope uptake and therapeutic response in metastases compared to primary tumors [72]. Furthermore, in some specific cancers such as anaplastic thyroid cancer that are resistant to radioactive iodine therapy due to loss of NIS expression, the use of MV-NIS in combination with radioactive iodine can be employed as an effective alternative approach, allowing the elimination of this resistance and destruction of cancer cells both in vitro and in vivo [73].

Due to the lack of efficient antibody response in myeloma patients, the probability of neutralization of MV-NIS particles by circulating antibodies is extremely lower among these patients than among the patients with other malignancies [56]. This result suggests that surviving the host immune response can play a decisive role in the outcome of treatment with MV-NIS. Accordingly, it has been shown that the route of MV-NIS administration is effective in escaping the immune system and treatment outcome. Therefore, compared to intravenous injection, intratumoral administration of MV-NIS has a more significant antitumor effect because of avoidance from the systemic immune response to measles [64]. Intratumoral MV-NIS therapy of human pancreatic cancer xenografts in mice resulted in a significant reduction in tumor volume and increased the survival time of the treated mice compared with the control mice [61]. However, the results of a study showed that there was no effective synergy between MV-NIS and ^131^I radiotherapy after the intratumoral injection of MV-NIS, which was clearly due to the lack of uniform distribution of MV-NIS infection within the BxPC-3 pancreatic tumors. It is while in stably NIS-expressing BxPC-3 tumors, rapid tumor regression occurred following administration of ^131^I [59].

To improve the function of MV-NIS particles, alternative methods are also under investigation. MV-NIS particles have a great potential for fusion-dependent cell-to-cell propagation, which in addition to the destruction of noninfected tumor cells also can considerably protect the virus from neutralizing antibodies [17]. Lethally irradiated MM1 cells have been shown to still be able to express MV genes and can transmit this infection to other myeloma cells in the presence of measles immune serum that can be attributed to the cell-to-cell spread of MV-NIS infection. Additionally, in mice bearing disseminated human myeloma, immunized with measles immune serum, administration of MV-NIS alone is not capable of inducing antitumor effect. However, lethally irradiated MV-NIS-infected MM1 cells can survive the passive antimeasles immunization and transmit MV-NIS infection to the cancer cells, leading to the destruction of tumor and increase in the survival of mice [74].

Co-administration of MV-NIS with other anticancer agents can be very helpful in progressing treatment and completing the treatment process. A study investigated the efficacy of MV-NIS for the treatment of ovarian cancer in athymic mice and showed that dual therapy with MV-CEA and MV-NIS has a better therapeutic effect compared to treatment with each virus alone [75]. In another survey, a combination of several therapeutic agents including MV-NIS, virally-directed ^131^I, External beam radiotherapy, and SAR-020106 (as Chk1 inhibitor) was used as a quadruplet regimen. According to the obtained data, these agents had a synergistic effect on each other, which can ultimately improve the therapeutic outcome of head and neck and colorectal cancers both in vitro and in vivo [76].

Along with continuous progress in MV-NIS-based preclinical studies, the next step is to conduct detailed and extensive clinical studies on patients with different cancers to tailor this method to therapeutic purposes. Patients with relapsing drug-refractory myeloma who received systemic administration of MV-NIS with radioiodine showed a good response to treatment [60]. In another clinical trial of MV-NIS for drug-resistant ovarian cancer therapy, the results indicated a significant increase in median overall survival among the patients. The results also showed that treatment with MV-NIS strongly stimulates the effector T cells against tumor antigens, leading to an effective antitumor cellular immune response [58]. A Phase I clinical trial employed MV-NIS alone or in combination with cyclophosphamide for targeted infection and extensive destruction of myeloma cells, and the results were highly satisfactory [57]. Incorporating findings from pre-clinical surveys into the clinical phase will gradually provide the basis for integrating therapeutic approaches to develop more advanced MV-NIS-based therapies.

### NIS tracking of tumor therapy

MV-NIS particles are not only used in the treatment of various cancers, but also in tracking the outcome of treatment and helping to achieve more accurate imaging of tumors and tracing cancer cells with contrast agent concentration inside the tumor. ^123^I or ^99^TcO_4_^−^ micro-single-photon emission CT/CT has been used for imaging the mice with BxPC-3 xenografts and showed a great tumor infection rate and radionuclide uptake. The results substantiated that accurate localization of MV-NIS infection through a noninvasive tumor tracking system can replace costly and time-consuming methods like autoradiography and immunohistochemistry [62]. In another study, human BxPC-3 pancreatic tumor xenografts in mice were imaged with micro-CT and micro-SPECT/CT after direct injection of contrast agent/MV-NIS mixture into tumors in order to track viral distribution within the tumor. This method allows accurate determination of the intratumoral viral distribution and real-time viral propagation and is appropriate for MV-NIS monitoring purposes [77]. Jung et al. reported that NIS expression using oncolytic MV can concentrate radioisotopes in infected cells in vitro and in vivo and the accumulation of radioisotopes can be quantitatively imaged by SPECT/CT. Their imaging results, both in vitro and in vivo, revealed a direct relationship between infectious virus particles and radioisotope uptake [78]. An intravital imaging system was successfully used by Kemler et al. in non-invasive imaging of tumor cells after injection of a fluorophore-expressing MV into the tumor. The fusogenic virus had a remarkably fast replication and developed numerous infection centers along with the formation of several syncytia [63]. Such methods enable the determination of in vivo distribution pattern and concentration sites of MV-NIS particles within the tumor tissue, helping to determine the dissemination of tumors and also the effectiveness of the treatment process.

### Conclusion

The ability to selectively transduce recombinant oncolytic viruses to the tissues of interest together with the emergence of molecular imaging techniques provide real-time quantitative information about both the pharmacokinetics and pharmacodynamics of viral therapy, allowing personalized treatment of cancers. With current advances in molecular imaging, it has been possible to visualize intratumoral infections in more detail and with higher resolution. Recently, NIS-mediated nuclear imaging has been rapidly developing for pre-clinical and clinical applications to optimize treatment parameters such as the viral dose, route of administration, and vector design. In this system, changing the biodistribution of a radioisotope at the site of reporter gene expression provides accurate insight into the viral evolution and locations of transgene expression over time. Collectively, genetically modified viruses along with molecular imaging techniques play crucial role in improving therapeutic outcomes and thus appear to be promising approaches for cancer therapy.

## Data Availability

Not applicable.

## Abbreviations

OVs: Oncolytic viruses
ICD: Immunogenic cell death
APCs: Antigen presenting cells
NK: Natural killer
MHC-I: Major histocompatibility complex class-I
CTL: Cytotoxic T-lymphocytes
PRRs: Pattern recognition receptors
SPECT: Single-photon emission computed tomography
OMV: Oncolytic measles virus
NIS: Sodium iodide Symporter
CPA: Cyclophosphamide
MSCs: Mesenchymal stem cells
CCD: Charge-coupled device
PET: Positron emission tomography
CT: Computed tomography
MRI: Magnetic resonance imaging
GFP: Green fluorescence protein
BLI: Bioluminescence imaging
TK: Thymidine kinase
AZT: Azidothymidine
hSSRT2: Human somatostatin receptor 2
hNET: Human norepinephrine transporter
MIBG: Metaiodobenzylguanidine
hNIS: Human sodium/iodide symporter
TPO: Thyroid peroxidase
THs: Thyroid hormones
cAMP: Cyclic adenosine monophosphate
MPNST: Malignant peripheral nerve sheath tumor
DSFC: Dorsal skinfold chamber
CPEs: Cytopathic effects
