# Correction to: Virotheranostics, a double-barreled viral gun pointed toward cancer; ready to shoot?

**DOI:** 10.1186/s12935-020-01611-2

**Published:** 2020-10-30

**Authors:** Mohsen Keshavarz, Ailar Sabbaghi, Seyed Mohammad Miri, Abolhasan Rezaeyan, Yaser Arjeini, Amir Ghaemi

**Affiliations:** 1 The Persian Gulf Tropical Medicine Research Center, The Persian Gulf Biomedical Sciences Research Institute, Bushehr University of Medical Sciences, Bushehr, Iran; 2 Department of Influenza and Other Respiratory Viruses, Pasteur Institute of Iran, Tehran, Iran; 3 Department of Virology, Pasteur Institute of Iran, Tehran, Iran; 4 Department of Medical Physics, School of Medicine, Iran University of Medical Sciences, Tehran, Iran; 5 Virology Department, School of Public Health, Tehran University of Medical Sciences, Tehran, Iran

## Correction to: Cell Int (2020) 20:131. https://doi.org/10.1186/s12935-020-01219-6

Following publication of the original article [1], we were notified the Department of Virology, Pasteur Institute of Iran should have been mentioned as the only affiliation for the third and last author. The revised affiliation is reflected in this correction article.
